# Supplementary material for: Factors associated with and socioeconomic inequalities in breast and cervical cancer screening among women aged 15–64 years in Botswana
Source: PLoS One. 2021 Aug 4;16(8):e0255581. doi: 10.1371/journal.pone.0255581 (PMC8336819; doi:10.1371/journal.pone.0255581)
Supplement: S1 File — (DOCX) [file pone.0255581.s001.docx]

**Explaining how the concentration index for the study was calculated**

The concentration index was defined as;

$$1. ∁=\frac{2}{n\mu h}\sum_{i-1}^{n} hiRi-1-\frac{1}{n}$$

Where: h*_i_* is the cancer screening variable, μ*_h_* is its mean, and R*_i_*=*i*/n is the fractional rank of individual *i* in the living standards distribution, with *i*= 1 for the poorest and *i*= n for the richest (O’Donnell, van Doorslaer, Wagstaff et al. 2008). The index summarizes information through the imposition of value judgments about the weight given to inequality at different points in the living standard distribution. The concentration index depends only on the relationship between cancer screening variable and the rank of the living standard variable (h*_i_*r*_i_*) and not on variation of living standard variable itself (Wagstaff, Bilge, Sajaia and Lokshin 2011). The value judgments implicit in the index are seen when the index is written as:

$$2. ∁=1-\frac{2}{n\mu}\sum_{i-1}^{n} hi(1-Ri)$$

The quantity h*i*/n*_μ_* is the ith person share of a specified cancer screening outcome. This is then weighted in the summation by twice the complement of the person’s fractional rank, that is, 2 (1–R*i*). So the poorest person has the share of a specified cancer screening outcome weighted by a number close to two. The weights decline in a stepwise fashion, reaching a number close to 0 for the richest person. The extended concentration index is then 1 minus the sum of these weighted health shares.

$$3. C\left( v \right)=1-\frac{v}{n\mu}\sum_{i-1}^{n} hi{(1-Ri)}^{(v-1)}$$

Where: *v*is the inequality-aversion parameter (the weight attached to the *i*th women’s cancer screening share), h*i*/nμ, is now equal tov(1–R*i*)(*v−1*), rather than by 2 (1–R*i*).When *v*= 1 everyone’s cancer screening is weighted equally. As *v* is raised above 1, the weight attached to the screening of a very poor person rises, and the weight attached to the health of a person above the55^th^ percentile decreases. Achievement Index (AI) was used to reflect average level of a specified health variable e.g. breast and cervical cancer screening and the inequality in screening between the poor and the better off. The index is defined as a weighted average of a specified cancer screening variable in the sample with higher weights attached to the poor than to better-off. The index is given as:

4. $1\left( v \right)=\frac{1}{n}\sum_{i-1}^{n} hiv{(1-Ri)}^{(v-1)}$

This index can be shown to be equal to:

$$1\left( v \right)=\mu\begin{matrix} [1-C\left( v \right)] \end{matrix}$$

When h is a measure of good health, high values of I*(v)* are considered good and C *(v)*> 0 (good health is higher among the non-poor). If a specified cancer screening variable declines monotonically with living standard, the greater is the degree of inequality aversion, and the greater is the wedge between the mean (*μ*) and the value of the index I (*v*) (O’Donnell, van Doorslaer, Wagstaff et al. 2008; Wagstaff, Bilge, Sajaia et al. 2011). Indirect method of standardization was used to reflect differences across socioeconomic groups while controlling other determinants of a specified cancer screening variable. The standardizing variables are those correlated with the living standard measure and that of the cervical and breast cancer screening outcomes from existing empirical literature. Such standardization provides a way to remove components of inequalities from socioeconomic related inequalities and describe the distribution of the health outcomes by socioeconomic status conditional on other demographic, socio-economic factors (Wagstaff, Bilge, Sajaia et al. 2011)

$$5. y=\propto+\sum_{j} BjXij+\sum_{k} ykZkj+\varepsilon i$$

Where:$y$*i* is the cancer screening variable for the *ith* woman; and α, βand$y$are parameter vectors, xj are confounding variables used to standardize, and zk are non-confounding variables for which we do not want to standardize but do want to control for in order to estimate partial correlations with the confounding variables.*α*,*βj* and *yk* parameter estimates of individual values of the confounding variables (x*ji*), and sample means of the non-confounding variables (zk) are then used to obtain the predicted values of the cervical and breast cancer screening indicator *γi*. Estimates of indirectly standardized cancer screening outcomes were then computed by the difference between actual and predicted outcomes plus the overall sample mean (O’Donnell, van Doorslaer, Wagstaff *et al*. 2008; Wagstaff, Bilge, Sajaia *et al.* 2011). Socioeconomic related inequalities were decomposed into the contributions of individual factors to wealth- related health inequality, in which each contribution is the product of the sensitivity of heath with respect to that factor and the degree of income-related inequality in that factor.

6. $\mathrm{Yi}=\propto\sum k\beta kXki+\epsilon i$

Where: Yi= 1 for the specified health variable, Xka set of exogenous determinants of that cervical and breast cancer screening variable andβk coefficient determinant Xk, and €I is random error term.
